# Heat, work and subtle fluids: a commentary on Joule (1850) ‘On the mechanical equivalent of heat’

**DOI:** 10.1098/rsta.2014.0348

**Published:** 2015-04-13

**Authors:** John Young

**Affiliations:** Hopkinson Laboratory, Engineering Department, University of Cambridge, Trumpington Street, Cambridge CB2 1PZ, UK

**Keywords:** conservation of energy, first law of thermodynamics, caloric theory, heat, work

## Abstract

James Joule played the major role in establishing the conservation of energy, or the first law of thermodynamics, as a universal, all-pervasive principle of physics. He was an experimentalist *par excellence* and his place in the development of thermodynamics is unarguable. This article discusses Joule's life and scientific work culminating in the 1850 paper, where he presented his detailed measurements of the mechanical equivalent of heat using his famous *paddle-wheel* apparatus. Joule's long series of experiments in the 1840s leading to his realisation that the conservation of energy was probably of universal validity is discussed in context with the work of other pioneers, notably Sadi Carnot, who effectively formulated the principle of the second law of thermodynamics a quarter of a century before the first law was accepted. The story of Joule's work is a story of an uphill struggle against a critical scientific establishment unwilling to accept the mounting evidence until it was impossible to ignore. His difficulties in attracting funding and publishing in reputable journals despite the quality of his work will resonate with many young scientists and engineers of the present day. This commentary was written to celebrate the 350th anniversary of the journal *Philosophical Transactions of the Royal Society*.

## 1. Introduction

**Figure 1.**
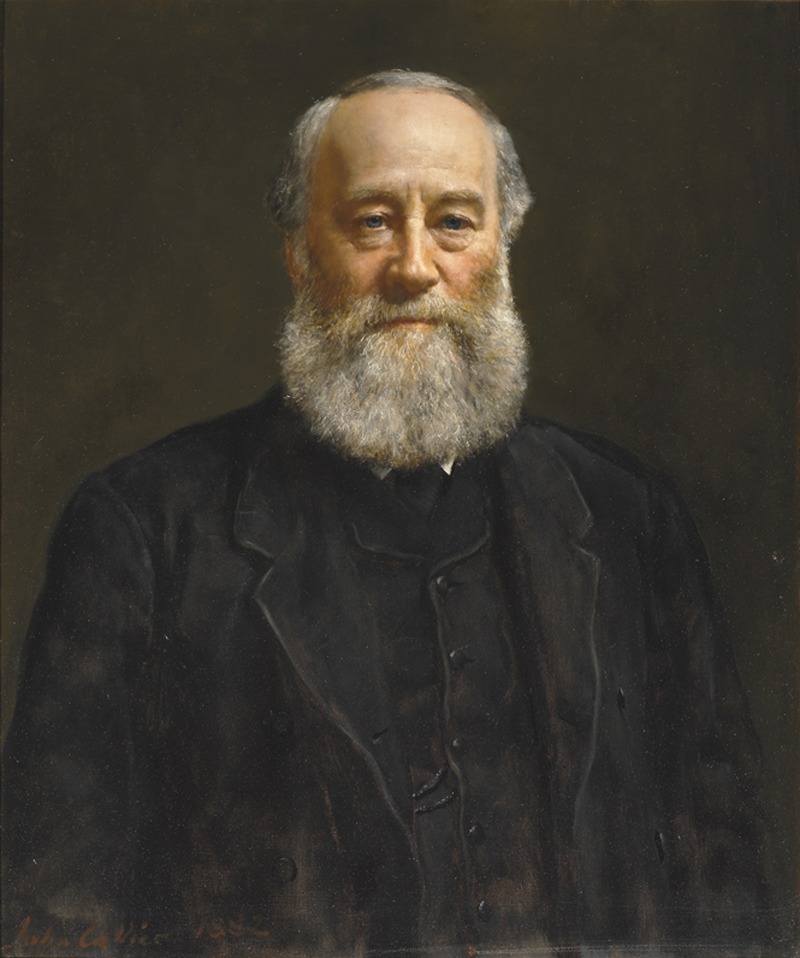
James Prescott Joule. Copyright The Royal Society.

Outside the scientific and engineering communities the name of James Prescott Joule (figure 1) is not widely known although virtually every packet of food purchased in a supermarket lists the energy value of the contents in the now standard SI unit, the *joule*. But old habits die hard and the superseded unit, the *calorie*, still finds greater favour with the general public.^1^ When, for example, did you last hear someone declare, ‘there were far too many joules in that pudding’? Until 1853, when William Rankine coined the terms *potential energy* and *actual* (i.e. kinetic) *energy*, Joule himself would not have been familiar with the word *energy* as a precisely defined scientific quantity. He would, however, have recognized calorie as being related to the *caloric theory of heat*, a theory that was accepted by almost all scientists of the time (then called *natural philosophers*) and which Joule was instrumental in overturning and replacing by the axiomatic principle we now call the *conservation of energy* or the *first law of thermodynamics*.

## 2. Joule's early life

James Joule (1818–1889) was born in Salford near Manchester, the heartland of the industrial revolution. In the words of the English experimental physicist Patrick Blackett, Salford was ‘one of those towns in which so much of the nation's wealth has been made and on which so little of it has been spent’. Nevertheless, Joule's family enjoyed a good life for they owned and operated a local brewery which became the largest in the region. They were prosperous and middle class, and they lived well in a substantial house with several servants.

In 1823, the family moved to Swinton nearby. The young James Joule was not a healthy child and a spinal weakness gave him a slight, though not pronounced, deformity. He did not attend school and was tutored at home where he made slow progress. Unsurprisingly, he was shy in company. Indeed, Joule was never able to command respect by the force of a strong personality and this may well account for his comparative obscurity outside the scientific community. He needed the support of someone who possessed the gifts he lacked, but it was not until 1847 that he found that person in the shape of William Thomson, later Lord Kelvin (1824–1907), who, although 6 years younger, had no trouble with self-publicity. Until then Joule struggled, publishing papers of major scientific importance but making almost no impact whatsoever.

The definitive account of Joule's life and work is the book by Donald Cardwell [1]. In Cardwell's description of Joule's formative years, he stresses the important influence of John Dalton (1766–1844), a local teacher and natural philosopher with radical scientific ideas who was an early proponent of the *billiard-ball* atomic theory of gases. From 1834 to 1837, James and his elder brother Benjamin studied under Dalton, receiving twice weekly sessions on arithmetic, geometry, chemistry and physics. Joule's fascination with experimental work started in this period but he did not acquire his obsession with experimental precision from Dalton. According to Humphry Davy, Dalton was a ‘very coarse experimenter’ and he often used rough and inaccurate instruments even when better ones were available. Nevertheless, Dalton was greatly celebrated in his own lifetime and on his death 40 000 people filed past his coffin as he lay in state in Manchester Town Hall, an astonishing accolade that almost certainly would have horrified the man himself who was quiet and retiring.

Given Joule's burgeoning scientific talents, it was a remarkable stroke of good fortune that a man of John Dalton's abilities was retained to tutor the two brothers. Dalton was a Fellow of the Royal Society and knew many of the leading scientists of the day. But he was also a scientifically independent thinker and it may well have been this characteristic which impressed the young Joule to such an extent that, when he was developing his own views about energy transformation, he was prepared to stand firm in print when almost every other natural philosopher in the world disagreed with him.

## 3. Physics in the early nineteenth century

Nowadays, it is difficult to empathize with the scientific and technical culture of the early nineteenth century. In Britain, no science degrees were awarded and there were no professional scientific qualifications. Only a small minority of those who published scientific papers were gainfully employed in science and Joule himself conducted most of his experiments in the cellar of his house as a private individual. However, the development of the steam engine, most notably by James Watt in the late eighteenth century, was stimulating an interest, particularly among engineers, in the fundamental principles of the technology.

Natural philosophy was divided into the *finished sciences* (Newtonian mechanics, planetary astronomy and optics) and the *progressive sciences* (botany, physiology, zoology, geology, chemistry, heat, electricity and magnetism). Electricity and heat were regarded as part of chemistry and thermal effects were thought to be due to the action of a *subtle fluid* called *caloric* which could be stored in bodies and transferred from one to the other.

The caloric theory of heat held sway throughout the late eighteenth and the first half of the nineteenth century. Virtually all natural philosophers accepted the concept that caloric could pass from one body to another by conduction and was conserved in the process. This theory was given convincing credibility in *The Analytical Theory of Heat* by the French mathematician and physicist Joseph Fourier (1768–1830) [2]. Fourier's treatise was a mathematical *tour de force* introducing, as it did, the solution of the heat conduction equation using what are now known as Fourier series to represent arbitrary functions which could even have discontinuities. Fourier claimed that, given the thermal properties, state and form of a body, he could predict its thermal state at any time in the future and had thus essentially completed the scientific study of heat. But, although the work was hugely influential, it was seriously restricted because it ignored the situation whereby heat was applied and mechanical work was performed in an engine. Given the huge success of the steam engine in powering the industrial revolution, this was a remarkable, indeed inexplicable, oversight on the part of Fourier and his contemporaries.

By the 1820s, few scientists had questioned the caloric theory. The most famous of the dissenters was the American military adventurer and physicist Benjamin Thompson (1753–1814), better known as Count Rumford. Rumford had a remarkable life. He fought in the American War of Independence, moved to London where he received a knighthood from King George III, and then spent nine years as the minister charged with re-organizing the Bavarian army. Nevertheless, it was his famous *cannon boring experiment* which has secured his place in history [3]. Rumford observed that the frictional heat generated by boring cannon in the arsenal in Munich was apparently limitless. To demonstrate this he immersed a cannon barrel in water and, using a specially blunted boring tool, found that the water boiled in under 3 hours. He then argued that this seemingly unlimited generation of heat was incompatible with the caloric theory and concluded that the only thing communicated to the barrel was motion.

Rumford himself did not attempt to calculate the so-called *mechanical equivalent of heat* (usually given the symbol *J*) and his description was essentially qualitative. However, Joule [4] made a rough estimate from the data in Rumford's original paper and concluded that, ‘the heat required to raise a lb. of water 1°F will be equivalent to the force represented by 1034 foot-pounds’. Before the mid-nineteenth century, the term *force* was often used to denote what we now call *work* (though clearly the unit foot-pound does not represent a force). The heat required to raise the temperature of 1 lb. of water by 1°F is now called a British thermal unit (Btu) and the currently accepted value for *J* is 778.0 foot-pounds per Btu. So, Rumford's data did give a value of the correct order of magnitude.

## 4. The contribution of Sadi Carnot

Before the industrial revolution most mechanical power was generated by animals (horses and oxen) or by waterwheels. The efficiency of a waterwheel could be calculated using Newtonian mechanics. The power input was obtained from the water flow rate and the height the water fell from the millpond, and the power output from the height that a known weight could be raised against gravity in a given time. The ratio of the two quantities gave the efficiency of the engine. Clearly, this type of analysis could not be used to calculate the efficiency of a steam engine.

The man who addressed this problem, and, in so doing, laid the foundations of the science of thermodynamics, was Nicolas Léonard Sadi Carnot (1796–1832). Sadi Carnot was a member of the French upper class and his father had been lucky to survive the excesses of the French revolution. Carnot the younger received a good education in mathematics and physics but this can hardly explain the sheer brilliance of the short book he published in 1824 [5]. Few copies were printed at the time and the publication was completely overlooked by Carnot's contemporaries. However, in fewer than 100 short pages Carnot provided an exposition of thermodynamics remarkably similar to the way it is taught to engineers today. *Reflections on the motive power of heat* should be on the reading list of all practising thermodynamicists. It is the work of a truly revolutionary scientific thinker who was able to describe his concepts with crystal clarity and impeccable mathematical logic.

On reading Carnot's memoir one is struck by his depth of understanding and the generality of his conclusions. It is all there! As a model of a steam engine, he conceived the generalized heat engine operating between a high-temperature heat source (the furnace) and a low-temperature heat sink (the condensing water). He then deduced that, for a given transfer of caloric, the maximum work output depends not on the working fluid, but solely on the temperatures of the heat reservoirs. He introduced the idea of *completing the cycle* (because a steam engine is an open circuit device) and further arguments led to the conclusion that the cyclic engine with maximum efficiency is one that can operate reversibly. Taking an ideal gas as the working fluid, he then calculated how the state of the fluid changes as it passes around the cycle. This was followed by a discussion of the principles in the context of real engines and the realization that whenever heat is transferred from one body to another across a finite temperature difference there will be a loss in the possibility for producing mechanical work.

Carnot's achievements were staggering but the story does not quite end there. An inveterate note maker, Carnot recorded his thoughts on all manner of topics. These notes have survived and in one he wrote, ‘When a hypothesis no longer suffices to explain phenomena, it should be abandoned. This is the case with the hypothesis which regards caloric as matter, as a subtle fluid’. He then proposed that heat was a form of motion of the *molecules* (meaning the constituent parts) of a body. He came to the conclusion that radiative heat must be a form of vibratory motion because of its similarity to light and wrote, ‘Could a motion (that of radiating heat) produce matter (caloric)? No, undoubtedly; it can only produce a motion. Heat is then the result of this motion. Then it is plain that it could be produced by the consumption of motive power, and that it could produce this power’.

Evidently, Carnot was on the verge of including a form of the conservation of energy in his exposition of thermodynamics. Sadly, however, he died of cholera in 1832 at the age of 36. Luckily, his memoir did not die with him for it was discovered by Émile Clapeyron who had it republished in 1834 6. Even then, it was ignored by the scientific community until it was rediscovered by William Thomson in the 1840s. Thomson realized the importance of the work and provided an account of the theory for the English-speaking reader [7]. However, he was not prepared to renounce the caloric theory and wrote, ‘To deny it would be to overturn the whole theory of heat, in which it is the fundamental principle’.

## 5. Joule's experiments in the 1840s

James Joule had no doubt that the caloric theory was fundamentally flawed. He arrived at this conclusion with growing conviction through a long series of experimental investigations starting in the late 1830s and extending through the 1840s. In fact, Joule's suspicions concerning the relation between heat and work may stem from an even earlier period. In 1813, the engineer Peter Ewart published a paper in the *Manchester Memoirs* [8] urging his readers to accept the principle of the conservation of *vis viva*. The term *vis viva* literally means *living force* and was defined as mass multiplied by velocity squared, i.e. twice the kinetic energy. Ewart wrote, ‘If we could get rid of all the imperfections in our steam engines we should find that a certain amount of heat always yielded a fixed equivalent of work’. The notable point is that John Dalton helped to write this paper and so it may have been through Dalton that Joule first learnt of this attempt to bring the conservation of energy into the picture.

The 1830s was a decade of great advances in the science and application of electricity. In 1831, Michael Faraday (1791–1867) discovered the phenomenon of electromagnetic induction and this was rapidly followed by the invention of the electric motor and generator, clear demonstrations of the inter-convertibility of mechanical and electrical work. The world was suddenly transfixed by the possibilities of electricity and James Joule was no exception. Most of his early experiments were electrically based and he acquired a laboratory stocked with electric batteries, electromagnets, motors, generators and galvanometers, most of which he made himself.

In 1839, Joule performed a series of experiments in which he discovered two electrical energy relationships, though he made no claim to originality. Firstly, he noted that the power output from his electric motor was proportional to the product of the current and the electromotive force. Secondly, he found that the heat produced by an electric current was proportional to the square of the current and the resistance of the wire (*Joule heating*) and was independent of the shape, size or form of the circuit. Some of this work was submitted to *Philosophical Transactions of the Royal Society* and was rejected. Joule later said [1, p. 41], ‘I was not surprised. I could imagine those gentlemen in London sitting round a table and saying to each other, “What good can come out of a town where they dine in the middle of the day?”.’

In the early 1840s, Joule applied his experimental expertise to investigate the generation of heat in electrical, chemical, mechanical and fluid systems. With this broader remit he began to realize that the results from a variety of quite different experiments were indicative of an underlying principle of much greater generality. The experiments involved measurements on electric generators and motors to calculate the mechanical equivalent of heat but the accuracy was not good and he obtained a variety of values between 587 and 1040 foot-pounds per Btu. He also devised an experiment in which water was heated by being forced through narrow tubes and obtained a value of 770 foot-pounds per Btu. When writing up this work he stated that he would lose no time in repeating and extending the experiments, ‘being satisfied that the grand agents of nature are, by the Creator's fiat, indestructible, and that wherever mechanical force is expended, an exact equivalent of heat is always obtained’ [1, p. 58]. This was an early qualitative statement of the conservation of energy, albeit backed up by some questionable religious evidence.

Despite the mediocre accuracy, Joule now suspected that he had discovered a universal constant of physics governing the conversion of work into heat and vice versa. He presented his results at a British Association meeting in Cork in Ireland [9] but the scientific community ignored his paper; it was still believed that Fourier had finalized the theory of heat in his treatise of 1822. Arguments supporting a kinetic theory of heat continued to appear sporadically but there was little support from the universities and influential institutions such as the Royal Society. In 1846, the scientific grandee Sir John Herschel, replying to John Waterston (a pioneer of the kinetic theory of gases), brushed him aside with, ‘I trust however that you will excuse me if I say that I have no time for the subject’ [1, p. 61].

Undeterred, Joule ploughed on. This time he investigated the changes in temperature produced by the expansion and compression of air. The experiments were carried out with two cylinders joined via a stopcock. One side was pressurized and the cylinders were placed in a water bath. When the valve was opened there was no change in the water temperature (because no overall work was done and the internal energy of the air remained the same) but further measurements showed that the cylinder subject to the expansion was cooled while that subject to the compression was heated by almost exactly the same amount. The experiments gave values for *J* of 820, 814 and 760 foot-pounds per Btu.

The paper [10] ended with the following bold statement:

The principles I have adopted lead to a theory of the steam-engine very different from the one generally received, but at the same time much more accordant with facts. It is the opinion of many philosophers that the mechanical power of the steam-engine arises simply from the passage of heat from a hot to a cold body, no heat being necessarily lost during the transfer. This view has been adopted by Mr. E. Clapeyron…. This philosopher agrees with Mr. Carnot in referring the power to vis viva developed by the caloric contained by the vapour, in its passage from the temperature of the boiler to that of the condenser. I conceive that this theory, however ingenious, is opposed to the recognised principles of philosophy, because it leads to the conclusion that vis viva may be destroyed by an improper disposition of the apparatus…. Believing that the power to destroy belongs to the Creator alone I entirely coincide with Roget and Faraday in the opinion that any theory which, when carried out, demands the annihilation of force, is necessarily erroneous. The principles, however, which I have advanced in this paper are free from this difficulty.

The paper was thus notable on four counts. Firstly, the values for *J* were similar to those from the electrical experiments. Secondly, in discussing the steam engine, Joule insisted that the heat rejected to the condenser was less than that coming from the boiler, the difference being converted into mechanical work. Thirdly, a religious piece of evidence was again quoted in support of the science. Fourthly, it is evident that by 1845 Joule was conversant with Carnot's work.

The paper was submitted to *Philosophical Transactions of the Royal Society* but the editorial board was again unimpressed, even by the citation of the Almighty. It was summarily rejected and was eventually published in the *Philosophical Magazine*, a more liberal journal [10].

In 1844 and 1845, Joule attended the annual British Association meetings but the scientific community was again indifferent, even hostile. Joule met the two men who dominated British physical science at the time, John Herschel and Michael Faraday. He later wrote to them, rather obsequiously, but they rejected his theory. Joule therefore decided to give a public lecture in Manchester. The lecture included a statement of the conservation of energy within the context of his experiments and the proposition that it would prove to be of entirely general application. Unfortunately, it was not a great success; he had to compete in the newspapers that week with a bad shipwreck and two dreadful murders!

Then came the British Association meeting at Oxford in 1847 when, against the odds, everything changed. Joule described his paddle-wheel experiment, a precursor to the much more elaborate paper [4] which is the subject of this article. He presented late in the day and the chairman asked him to confine himself to a brief summary—how many of us have heard, with sinking heart, that same request when presenting at a conference? Afterwards, Joule said that the paper would have passed unnoticed except for a young man at the back who stood up and asked some penetrating questions. The young man was William Thomson—though Thomson later denied Joule's account, saying he remained seated and put his questions after the meeting!

The relationship between Joule and Thomson flourished, although Thomson was far from being a convert. He wrote to his father, ‘Joule is, I am sure, wrong in many of his ideas but he seems to have discovered some facts of extreme importance, as for instance, that heat is developed by the friction of fluids’. He also sent Joule's papers to his brother James Thomson saying that they would astonish him though he felt there must be ‘some great flaws’. In reply, James wrote, ‘Some of Joule's views have a slight tendency to unsettle one's mind as to the accuracy of Clapeyron's principle. If some of the heat can absolutely be turned into mech. eff. Clapeyron may be wrong’.

## 6. The paddle-wheel experiment

**Figure 2.**
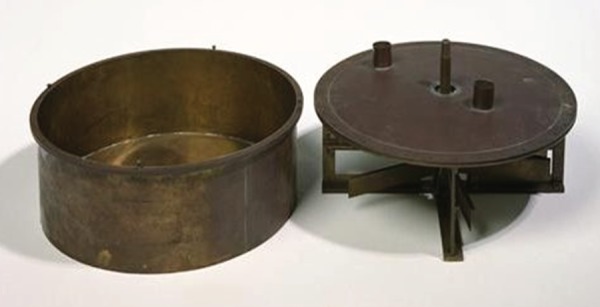
One of Joule's original paddle-wheel assemblies. (Courtesy of the Science Museum, London.)

Joule's paddle-wheel experiment [4] is the most famous of his conservation-of-energy experiments because, as we now know, it gave the most accurate results for the mechanical equivalent of heat. The experiment was performed in the cellar of Joule's house and was simplicity in itself (see figs. 1–9 of [4]). A cylindrical vessel containing water or mercury was fitted with a paddle-wheel which rotated between fixed vanes on a vertical axis. The paddle-wheel was driven by two strings, wound round the shaft, which passed over two pulleys and were attached to falling weights. The work input was calculated as the product of the weight and the distance of fall to the floor of Joule's cellar. The heat generated was obtained from the temperature rise of the water or mercury (and the associated metalwork). In order to obtain a measurable increase in temperature of 0.5–2°F, the weights were wound back up and the experiment was repeated some 20 times as quickly as possible. Several paddle-wheel assemblies were constructed using different materials and figure 2 shows a photograph of the one that is housed in the Science Museum in London.

In the introduction to the paper Joule mentions previous work on the dynamical theory of heat, including Rumford's cannon boring experiment. He then turns to a description of the experimental apparatus, starting with a discussion of the accuracy of the thermometers used to measure the temperature rise of the water or mercury. The success of the experiments depended on being able to obtain accurate measurements of temperature changes of the order of 1°F or less and Joule used a number of specially made thermometers. He claimed that, with practice, he could read the thermometers with the naked eye to one twentieth of a division, corresponding to about 0.005°F. At risk of criticizing the great experimentalist, this does seem a trifle optimistic.

After describing the apparatus, Joule goes into a very detailed explanation about the various corrections made to the measurements to take account of heat loss and other possible errors. Clearly, this part of the analysis was crucial and had to be well documented but it does make turgid reading and Joule does not spare us the intricate details. We are then, in tables I–VIII, treated to a presentation of almost every single measurement he took, and every calculation he made. Nothing is omitted and, from the paper, it is possible to reproduce the experimental minutiae of all the tests. Although rather tedious, this is a lasting tribute to Joule's care and precision, and can be contrasted with modern-day technical publications where crucial data are often omitted, either deliberately or accidentally, or because of a journal page restriction policy.

Joule carried out five series of experiments to determine the mechanical equivalent of heat and the final results are listed in table IX of the paper. Series 1 involved a brass paddle-wheel rotating in a copper cylinder containing water and gave an average value of 772.692 foot-pounds per Btu. Series 2 and 3 used a wrought-iron paddle-wheel in a cast-iron cylinder containing mercury and gave averages of 772.814 and 775.352, respectively. Series 4 and 5 also used mercury as the fluid but involved friction between solids with the paddle-wheel replaced by a cast-iron wheel rotating in contact with a stationary wheel. The average values for these tests were 776.045 and 773.930. The consistency of the results was certainly remarkable, although one feels that Joule was more than a little optimistic in quoting six significant figures!

Joule considered the experiments in Series 1 to be the most reliable and the paper closes with two conclusions:

(1) that the quantity of heat produced by the friction of bodies, whether solid or liquid, is always proportional to the quantity of force expended;
(2) that the quantity of heat capable of increasing the temperature of a pound of water (weighed *in vacuo*, and taken at between 55° and 60°) by 1°F requires for its evolution the expenditure of a mechanical force represented by the fall of 772 lb through the space of one foot.

And that's it! Where, we ask, is the proposition about the conservation of energy and the relation between heat and work that is surely warranted by such careful experiments and consistent results? The answer is that Joule originally included a third conclusion but was obliged to remove it following the peer review process of the Royal Society editorial board [11]. We know this because, when the paper was reprinted with Joule's other scientific papers in 1883, he added a footnote stating that, ‘A third proposition, suppressed in accordance with the wish of the committee to whom the paper was referred, stated that friction consisted in the conversion of mechanical power into heat’.

Astonishingly, the original referee's report still survives because it was enclosed in a letter that Joule sent to Thomson in 1855 with the comment, ‘There are one or two passages in it which will, I dare say, amuse you’ [12]. The report is anonymous but the handwriting has been identified as none other than that of Michael Faraday! Faraday praised Joule's experimental skill and was happy to accept the first two conclusions. However, he then wrote, ‘It cannot nevertheless but be regretted as a conspicuous defect in his paper that, with the statement of this result and of the inductive process by which the Author has arrived at it, he has mingled up the statement of another proposition which appears to be deduced most illogically from it, to the effect that, because a given amount of heat when evolved by mechanical force requires always the same amount of force (measured as work) to evolve it, therefore heat is convertible into force and force into heat’.

Faraday did not really understand Joule's ideas about the dynamical theory of heat and he certainly was not going to allow them to appear in *Philosophical Transactions of the Royal Society* if he had anything to do with it. Of course, it is true that Joule had not presented any actual *proof* that work was converted directly into heat but, nonetheless, Faraday chose to ignore the mounting evidence presented in the series of papers published through the 1840s. Furthermore, his comment begs the question as to how exactly the conservation of energy could be established other than by experiments such as those devised by Joule. Be that as it may, Joule, like so many other scientists before and after, desperately wanted to get his work published in a top journal and so he compromised his beliefs and removed the offending statement. Shortly afterwards, he sent George Stokes, the physicist and mathematician, a copy of the paper and explained why he had withdrawn the third conclusion, adding, ‘I think this view will ultimately be found to be the correct one. I cannot see why it may not be as well asserted that the heat evolved is the cause of the loss of force in friction as vice versa’.

## 7. The legacy

The rest, as they say, is history—and it all happened very quickly. In the same year, 1850, Rudolf Clausius (1822–1888) published a paper showing how a slight alteration to Carnot's theory could bring it into line with Joule's demonstration that heat and work are interchangeable (see [13]). All that was required was to take Carnot's reversible cycle and assume that a proportion of the heat supplied from the hot reservoir was converted into work while the rest was rejected to the cold reservoir. In later papers, Clausius went on to state the second law of thermodynamics in one of the forms still taught to engineers and, most importantly, introduced the concept of entropy to quantify irreversibility and dissipative processes.

William Thomson, who had been so instrumental in supporting and promoting Joule (while not quite believing in the interchangeability of heat and work), was still making statements about the caloric theory in 1852. Thomson has probably received more credit than he deserves for his part in formulating the fundamentals of thermodynamics. It is true that, after discovering Carnot's work, he deduced the existence of the absolute thermodynamic temperature scale but his real expertise was in clarifying and reformulating the work of the pioneers. It is noteworthy that Thomson took no part in the development of the kinetic theory of gases in the second half of the nineteenth century. Here, the baton passed from Joule to Clausius and then on to Maxwell, Boltzmann and Gibbs.

With the overthrow of the caloric theory and the vindication of Joule's conservation principle one might have expected to observe the collapse of a large number of elderly scientific stout parties. This, of course, did not happen. As Max Planck later said, ‘A new scientific truth does not triumph by convincing its opponents and making them see the light, but rather because its opponents eventually die, and a new generation grows up that is familiar with it’. Nevertheless, no one wants to miss a bandwagon and only 2 years later, in 1852, the Royal Society, with scarcely a glance over its shoulder at its treatment of Joule's submissions, awarded him a Royal Medal for his paper on the mechanical equivalent of heat. But he still had to wait until 1870 before the Society was sufficiently convinced to award him the prestigious Copley Medal ‘for his experimental researches on the dynamical theory of heat’. Well, it would not have wanted to make a mistake!

In the 50 years following Joule's determination of the mechanical equivalent of heat, a number of scientists attempted to improve the precision of the result. These attempts met with mixed success until 1897 when Osborne Reynolds and his collaborator W. H. Moorby published the results of a series of experiments conducted at Owens College in Manchester where Reynolds was professor. The method chosen was to couple a water brake to a steam engine and measure the work dissipated by the brake in raising one pound of water from freezing temperature to boiling temperature. The care with which the experiments were performed was truly astonishing and Reynolds used his Bakerian Lecture of 1897 to describe the investigation. It was subsequently published as a mammoth 121 page paper in *Philosophical Transactions of the Royal Society*, every minute detail of the experiments being included [14]. The final value obtained was 776.94 foot-pounds per Btu, within 0.2% of the currently accepted value. In his obituary of Reynolds for the Royal Society, Horace Lamb [15] wrote that ‘the whole investigation is a model of scientific method and may claim to rank among the classical determinations of physical constants’.

The importance of the scientific legacy of James Joule's work is difficult to overstate. Over the century following his experiments classical thermodynamics was developed and honed until finally, with the work of Joseph Keenan and his colleagues at the Massachusetts Institute of Technology after the Second World War, it became the mature formalized science that it is today [16]. The kinetic theory of gases, developed by Clerk Maxwell and Ludwig Boltzmann, together with the statistical mechanics of Willard Gibbs, provided the means of interpreting the classical concepts of energy and entropy at a molecular level and work in these fields continues to this day.

James Joule's conservation of energy has become one of the bedrocks of technical analysis and, were he alive today, he could justifiably be very proud of his great heritage. It would be invidious to single out specific applications because its use is ubiquitous. Around the world, thousands of scientists and engineers use it every day for performing technical calculations, usually without giving any thought whatsoever to its validity, which is now simply taken for granted. Back in the 1840s, it was an altogether different story—but a very good story, nonetheless!

## Footnotes

1 In fact, the energy content of food is always quoted in kilojoules or kilocalories, the joule and calorie being very small quantities in this context. Common discourse tends to ignore the factor of 1000!

## Author profile

**John Young** was educated at King Edward's School, Birmingham, and Christ Church, Oxford, where he read engineering science. His PhD work was undertaken at the University of Birmingham and was concerned with non-equilibrium wet-steam flows in turbines. He then moved to the Whittle Laboratory at the University of Cambridge, where he continued this work and became involved with other aspects of turbomachinery research. In 1998 he became the Hopkinson and ICI Professor of Applied Thermodynamics at Cambridge. His research interests include the theory of non-equilibrium two-phase flow, wet-steam turbine technology, homogeneous nucleation theory, solid particle transport and deposition in turbulent gas flows, clean-coal technology, the thermodynamics of advanced power cycles, gas turbine blade cooling, multicomponent diffusion theory and solid oxide fuel cell technology. Without James Joule and the conservation of energy he would not have had a career.
